# Machine learning-based QSAR and molecular modeling of phytocompounds in *Barleria buxifolia L.* as a potential aldose reductase inhibitor

**DOI:** 10.3389/fbinf.2026.1766339

**Published:** 2026-03-03

**Authors:** Radul R. Dev, Anjana C. Lalu, Sinana Zarin, Bristow Ben Joseph, Rajesh Raju, Abhithaj Jayanandan, Sangeeth Thekkan

**Affiliations:** Centre for Integrative Omics Data Science (CIODS), Yenapoya (Deemed to be University), Mangalore, India

**Keywords:** aldose reductase, antidiabetic, Barleria buxifolia L, molecular docking, molecular dynamics, toxicity prediction

## Abstract

**Introduction:**

The traditional medicinal plant *Barleria buxifolia L* is well-known for its pharmacological properties. This study aims to predict the binding affinity of bioactive compounds obtained from *B. buxifolia* towards significant molecular targets associated with diabetes mellitus. The polyol pathway enzyme aldose reductase is associated with diabetes, making it a possible therapeutic target.

**Methods:**

By redocking the co-crystallised ligand sulindac sulfone into the aldose reductase binding site, the researchers proved the docking approach’s reliability with a 0.117 Å root mean square deviation (RMSD). Virtual screening showed that 9-Carbomethoxy-6,11-dichloroxy-5-oxoxantho [3,2-g] tetralin (9CDOT) as a promising inhibitor, surpassing sulindac sulfone, which had a glide score of −9.95 kcal/mol and binding energy of −55.10 kcal/mol.

**Results:**

The chosen molecule exhibited significant binding affinity through π–π interactions with Trp111 and Trp219, a stabilising hydrogen bond with Leu300, and hydrophobic contacts with Val297, Ala299, and Leu300. Halogen bonding connections were found with one chlorine facing Trp219, suggesting π-halogen interaction, and the other towards Leu300 and Leu301, indicating halogen-hydrophobic stabilisation. Synergistic interactions increase ligand target site affinity and specificity. The compound was non-hepatotoxic, non-neurotoxic, and non-cytotoxic, according to the toxicity prediction. Our investigation shows that 9CDOT, a phytocompound from *B. buxifolia*, strongly inhibits human aldose reductase.

**Discussion:**

The molecule is considered to be one of *B. buxifolia’s* active antidiabetic principles, making it a promising aldose reductase inhibitor lead candidate. Further verifying its potency, an AI-assisted PCA–PLS QSAR model also showed high predictive performance (R2 = 0.692), with 9CDOT showing a projected pIC_50_ of 7.52 (IC_50_ = 30 nM). To determine its therapeutic efficacy and investigate its potential as a lead candidate for the management of diabetes complications, more experimental validation is required.

## 1 Introduction

Diabetes has become a major global health concern due to rapid modernization and lifestyle changes. Globally, this disease is becoming more common, with developing nations like China and India having a significantly greater burden. In terms of the prevalence of diabetes among people aged 20 to 79, India is the second most affected country in the world, after China. In 2019, there were 77 million cases, and by 2030 and 2045, that number is expected to rise to roughly 101 million and 134.2 million, respectively (Pradeepa and Mohan, 2021). Diabetes-related mortality and economic loss have increased in tandem with the fast growth in diabetes prevalence (Chaudhary et al., 2021).

A chronic, complex, and polygenic metabolic disease, diabetes mellitus is characterized by insufficient insulin secretion, inadequate insulin sensitivity, or both. It can be difficult to categorize people with diabetes because the type is frequently based on the circumstances at diagnosis. Type 1 diabetes mellitus (T1DM), Type 2 diabetes mellitus (T2DM), gestational diabetes mellitus (GDM), neonatal diabetes, maturity-onset diabetes of the young (MODY), and secondary forms resulting from endocrinopathies or medication exposure are among the several kinds of diabetes mellitus (DM). The two main subtypes of these are T1DM and T2DM. While T2DM is characterized by increasing β-cell malfunction, frequently accompanied by insulin resistance, T1DM is caused by autoimmune destruction of pancreatic β-cells, resulting in decreased insulin production. While T2DM primarily affects middle-aged and older adults, T1DM usually appears in children and teenagers. It is frequently associated with long-term hyperglycemia caused by food and lifestyle choices (Tegegne et al., 2024; Sapra and Bhandari, 2023).

Diabetes mellitus (DM) is influenced by a number of interconnected factors. Genetic predispositions, environmental factors, lifestyle choices like inactivity, poor diet, smoking, and excessive alcohol consumption, as well as metabolic and hormonal imbalances like impaired β-cell function, insulin deficiency, hyperinsulinemia, and dysregulation of glucagon activity, are important contributors (Wu et al., 2014; Sami et al., 2025). Diabetes mellitus (DM) is characterized by symptoms that develop gradually and are mostly caused by persistent hyperglycemia. In addition to unexplained weight loss, the cardinal clinical characteristics include polyuria, polyphagia, and polydipsia. Other common symptoms include impaired vision, chronic fatigue, recurring infections, delayed wound healing, and itching (American Diabetes Association, 2010; Ramachandran, 2014). Oral hypoglycemic medications are frequently used for an extended period of time to treat diabetes and other chronic metabolic diseases. Long-term usage of these drugs, however, may be linked with adverse side effects and the emergence of drug resistance. In order to develop new antidiabetic treatments with better safety profiles and increased therapeutic efficacy, there is an urgent need for continued research (Sugandh et al., 2023; Chaudhury et al., 2017). In this regard, molecular markers linked to antioxidant defense and oxidative stress are important in the pathophysiology of type 2 diabetes mellitus. Diabetic individuals have been found to have elevated levels of malondialdehyde (MDA), nitric oxide (NO), and reactive oxygen species (ROS) as well as decreased activity of antioxidant enzymes such glutathione (GSH), catalase (CAT), and superoxide dismutase (SOD) (Jasvinder et al., 2022; Sabitha et al., 2024).

Aldose reductase (AR) (EC: 1.1.1.21) is a monomeric, cytosolic, NADPH-dependent enzyme of the aldo-keto reductase superfamily that catalyzes the first step in the polyol pathway, which converts glucose to sorbitol. Under normal glucose homeostasis, aldose reductase contributes minimally to glucose metabolism. However, during hyperglycemia, the flux of glucose via the polyol pathway leads to sorbitol accumulation in tissues. Sorbitol accumulation induces osmotic pressure and leads to osmotic stress. Excess NADH causes an increase in reactive oxygen species production inducing oxidative stress, leading to diabetic pathogenesis and its complications such as retinopathy, neuropathy, and nephropathy. Elevated fructose, on the other hand, contributes to cell injury. Based on sequence identity, aldo-keto reductases (AKRs) have been grouped into distinct families, AKR1 to AKR15; each family contains multiple subfamilies. The AKR1 family has been divided into five subfamilies, A to E. Of these, AKR1B is the most studied subfamily because of the potential role of its founding member, aldose reductase (AKR1B1), in the development of diabetic complications. Under normal glucose levels, aldose reductase plays only a minor role in metabolism because of its low affinity for glucose. However, during hyperglycaemia, its activity increases markedly, leading to sorbitol accumulation and contributing to diabetic complications (Singh et al., 2021; Thakur et al., 2021). Aldose reductase plays a central role in the development of diabetic complications, as it is the rate-limiting enzyme of the polyol pathway. Hyperglycemia has been shown to upregulate AR in tissues both *in vitro* and *in vivo*, and treatment with AR inhibitors mitigates hyperglycemia-induced vascular smooth muscle cell (VSMC) hyperplasia, hyperproliferation, and diabetic cardiomyopathy. Conversely, transgenic overexpression of AR in mice exacerbates diabetic cardiomyopathy, highlighting its pathogenic role. Although several AR inhibitors (ARIs) have been explored, their clinical efficacy remains limited, and side effects are a concern. Therefore, targeting aldose reductase continues to be a critical focus for developing safer and more potent therapies to prevent diabetes-associated metabolic and vascular complications (Singh et al., 2021).

The use of natural products, especially medicinal plants, in pharmaceutical development has gained increasing prominence in several developing countries. In recent decades, about 80% of individuals in developing countries have used medicinal herbs to treat various ailments safely and effectively. Phytochemistry has consequently gained considerable attention for the identification of bioactive compounds from medicinal plants (Gangaram et al., 2021; Lei et al., 2023; Am and Em, 2021). GC-MS and molecular docking are complementary methods frequently employed in drug development and natural product research. These methods expedite the identification of drug-like compounds and provide insights into drug-receptor interactions, facilitating precise binding at the target sites (Al-Raddadi et al., 2025; Durhan et al., 2022). In silico studies have shown the potential of bioactives from *Stigma maydis,* fenugreek, *Ocimum cufodontii, Dalbergia sisso*, demonstrating that plant derived compounds can interact effectively with diabetes related proteins (Durhan et al., 2022; Luo et al., 2023; Aliye et al., 2021; Vijh and Gupta, 2024). The genus *Barleria* (Acanthaceae) is widely used in Ayurvedic medicines and contains flavanoids, phenols, tannins, steroids, terpenoids, and cardiac glycosides. Several species, including *B. pratensis, B. lupulina*, *B. prionitis, B. dinteri, have demonstrated* antidiabetic, cytoprotective, and immunoprotective properties (Haridas et al., 2017; Suba et al., 2004; Sudheer and Praveen, 2021). The present study focuses on *Barleria buxifolia,* traditionally used by various ethnic communities. In this study, the antidiabetic potential of phytoconstituents previously characterized from *Barleria buxifolia* using GC-MS analysis (Selvi et al., 2017) was systematically explored. These bioactive compounds, reported to possess diverse pharmacological properties such as anti-inflammatory, antiulcer, antihypertensive, antiviral, antiobesity, antidiabetic, cardioprotective, vasoprotective, spasmolytic, respiratory analeptic, and carminative effects, were subjected to molecular modeling studies. Their possible inhibitory interactions with aldose reductase, a crucial therapeutic target involved in the pathophysiology of diabetes complications, were evaluated using the computational approach.

## 2 Methodology

### 2.1 Data extraction

The three-dimensional (3D) X-ray crystal structure of human aldose reductase was acquired from the Protein Data Bank (PDB). The retrieved structure was complexed with Sulindac Sulfone (pdb id: 3RX2) with a resolution of 1.90 Å (Zheng et al., 2012). For screening against the target, thirty bioactive compounds from *Barleria buxifolia L* were obtained from a previously published GC-MS analysis (Selvi et al., 2017).

### 2.2 Protein preparation

Protein Preparation wizard in the Schrödinger suite was used to prepare the target structure. Several steps are involved in the preparation workflow to ensure the accuracy and integrity of the target protein. In order to maintain the active site conformation, the NADPH cofactor was kept while structural artifacts, undesired ligands, crystallographic water molecules larger than 5 Å were eliminated, hydrogen atoms were added, bond orders were assigned, and side chains were added or optimized. In order to reduce steric conflicts and ensure proper geometry, the hydrogen bonding network was improved and the structure was energy minimized using the OPLS_4 force field, preparing it for docking and simulations.

### 2.3 Ligand preparation

As the 3D structures of the 30 bioactive compounds were unavailable, they were sketched using a 2D Sketcher tool (Supplementary Table S1). The generated structures were subsequently processed using the LigPrep module to obtain energetically minimized and chemically accurate 3D conformations. The ligands were desalted, and their possible tautomeric, stereoisomeric, and ionization states were generated using the Epik module at pH 7.0 ± 1.0. The OPLS_4 force field was used to minimize the energy of all ligand structures in order to improve molecular geometry and assign adequate atomic charges, thereby high-quality ligand conformations that are appropriate for molecular docking and molecular dynamics simulations.

### 2.4 Grid generation

The Schrödinger suite’s Receptor Grid Generation module was used to generate a 10 × 10 × 10 cubic grid box. The center of the grid was defined based on the coordinates of the co-crystallized ligand, sulindac sulfone, thereby encompassing the active-site region of aldose reductase. Sulindac sulfone, a non-steroidal anti-inflammatory drug with strong aldose reductase inhibitory action, was used to validate the binding site prior molecular docking studies were carried out. The co-crystallized ligand sulindac sulfone was used as a reference to create the receptor grid in the Schrödinger suite (Zheng et al., 2012).

### 2.5 Preparation for redocking

Using the Schrödinger suite, the co-crystallised ligand was extracted from the target protein structure and re-docked into the same binding site to validate the docking protocol (Henna et al., 2025). The protein preparation was done by eliminating all the water molecules, heteroatoms and ions, subsequently adding hydrogen atoms and assigning the appropriate bond ordering. Energy minimization was used to optimize the ligand, and the proper protonation states were assigned at physiological pH.

### 2.6 Virtual screening and estimation of binding free energy of top-performing ligands

The Schrödinger suite’s GLIDE module was used to generate a virtual screening workflow against aldose reductase and screen for ligands (Reddy et al., 2025). The QikProp module was used to analyze the drug-likeness of all phytocompound’s ADME characteristics prior to docking. The virtual screening workflow was executed in a hierarchical manner, comprising High-Throughput Virtual Screening (HTVS), Standard Precision (SP) docking, and Extra Precision (XP) docking (Dogga et al., 2022). Following each docking step, compounds were screened, and the molecules with the highest docking scores were chosen for further analysis. Further binding free energy calculations were carried out using the MM-GBSA (Molecular Mechanics-Generalized Born Surface Area) approach, which is implemented in the Prime module of the Schrödinger suite with the OPLS4 force field, based on the XP docking data. Based on their combined docking scores and MM-GBSA binding free energy values, the most promising molecules were identified, and the top-scoring ligand was subjected to further investigations (Gangadharan et al., 2024).

### 2.7 Toxicity prediction using ProTox3 webserver

Drug toxicity of the selected compounds was determined using the ProTox3 webserver. The server predicts multiple endpoints including hepatotoxicity, neurotoxicity, nephrotoxicity, respiratory toxicity, cardiotoxicity, carcinogenicity, immunotoxicity, mutagenicity, cytotoxicity, blood-brain barrier permeability, ecotoxicity, clinical toxicity, and nutritional toxicity. The compounds were submitted in SMILES format and ProTox3 predicted their lethal doses, and toxicological profiles based on machine-learning models (Banerjee et al., 2024).

### 2.8 Molecular dynamics simulation

Molecular Dynamics (MD) simulations of 200 ns were performed for both the standard and the inhibitor using the Desmond module from the Schrödinger suite to assess the stability of the ligand-bound complex. The simulation system was built using a system builder, with TIP3P as the solvent model and a 10 × 10 × 10 Å orthorhombic box under the OPLS4 force field. Appropriate numbers of 32 Na^+^ and 26 Cl^-^ ions were added to neutralize the system. The system was maintained at 300 K temperature and 1.013 bar pressure using a Nose–Hoover chain thermostat and a Martyna-Tobias-Klein barostat under NPT ensemble conditions. Following the simulation, Root Mean Square Deviation (RMSD), Root Mean Square Fluctuation (RMSF) analysis were performed for the apoprotein, standard complex, and the inhibitor complex to evaluate structural stability, residue-level flexibility, and correlated motions. The thermal_mmgbsa.py script was used to compute the post-MD simulation binding energy.

An analysis of the Free Energy Landscape (FEL) was carried out using the first two PCA components. The Boltzmann relationship was used to convert the probability densities of conformational states to relative free energy. A minor constant was included to prevent computational problems caused by zero probabilities. The data was smoothed using a Gaussian filter, which provided a thermodynamic view of the relative stability and preferred conformations of the protein and protein-ligand complexes as well as a clear visualization of energy basins and barriers.

### 2.9 Molecular bioactivity QSAR modeling using machine learning and an integrated PCA–PLS regression pipeline

The QSAR model was built using the dataset of aldose reductase from BindingDB with ligand SMILES and their IC50 value. Molecular structures, encoded as SMILES strings, were converted to RDKit molecule objects, and compounds with invalid structures or non-positive IC_50_ values were excluded. After being converted to molar units, IC_50_ measurements were standardized and converted into pIC_50_ values. The molecular descriptors were computed using RDKit and the descriptors with entire values as zero were eliminated to create a reliable model. After standardizing the resulting descriptor matrix and applying PCA to reduce dimensionality while maintaining 95% of the variance, PLS regression with up to five latent components was used to characterize the link between biological activity and molecular characteristics. Coefficient of determination (R^2^), Root Mean Square Error (RMSE), Mean Absolute Error (MAE), Mean Absolute Percentage Error (MAPE), Root Mean Squared Logarithmic Error (RMSLE), mean error, and maximum error were among the metrics used to quantitatively evaluate the model’s performance on the training set (Eriksson et al., 2006; Norinder, 1993). Finally, the fully trained pipeline, including scaling, dimensionality reduction, and regression steps, along with the selected descriptor set, was serialized for future predictive applications, enabling reproducible and efficient machine learning–based QSAR modeling.

## 3 Result

### 3.1 Validation of the docking protocol

**FIGURE 1:**
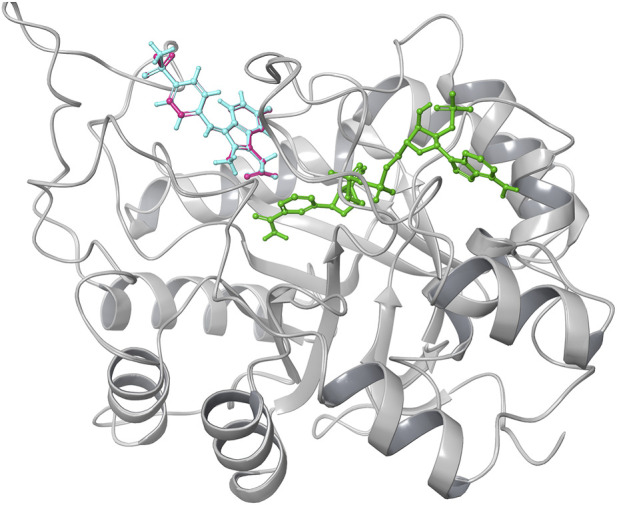
Validation of docking protocol done by re-docking Sulindac sulfone. The crystallographic structure of sulindac sulfone shown in (cyan) while the re-docked sulindac sulfone represented in (pink).

To validate the docking protocol, the co-crystallised ligand sulindac sulfone was re-docked into the Aldose Reductase binding site (Figure 1). The RMSD between the experimental and redocked ligand poses was calculated using heavy-atom superimposition in Maestro. The obtained RMSD value of 0.117 Å indicates an excellent overlap between the two conformations, confirming that the docking parameters accurately reproduce the experimentally observed binding mode.

### 3.2 Virtual screening of phytocompounds

The predicted pharmacokinetic and drug-likeness profiles of the top screened phytocompounds evaluated using the Qikprop module satisfied key ADME parameters, including optimal lipophilicity, permeability, oral absorption, and compliance with Lipinski’s Rule of five (Supplementary Table S2). These results indicate favourable pharmacokinetic properties and support the suitability of the selected compounds for further molecular docking studies.

**FIGURE 2:**
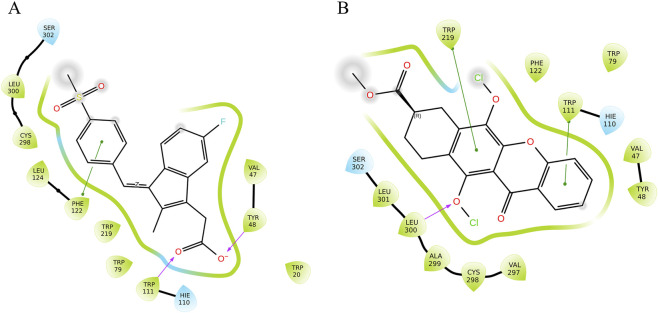
Ligand interaction diagram of Aldose reductase with **(A)** Sulindac sulfone, **(B)** 9CDOT.

Molecular docking analysis demonstrated multiple molecular interactions, including prominent hydrophobic interactions, hydrogen bonds and *π*-*π* stacking interactions with key residues within the aldose reductase active site. The molecular docking analysis of standard inhibitor sulindac sulfone, revealed a glide score of −9.95 kcal/mol and binding energy of −55.10 kcal/mol (Table 1), confirming its stable interaction within the aldose reductase active site. The Aldose Reductase and sulindac sulfone (standard) engaged in one π–π interaction with Phe122 and formed two hydrogen bonds with Tyr48 and Trp111. Virtual screening identified that 9-Carbomethoxy-6,11-dichloroxy-5-oxoxantho [3,2-g] tetralin (9CDOT) exhibited a favourable glide score of −8.492 kcal/mol and a binding energy of −69.63 kcal/mol as a top performing compound (Table 1). The more favourable binding energy of 9CDOT suggests stronger stabilizing interactions and enhanced binding potential compared to the reference inhibitor. Dual π–π stacking interactions contribute significantly to anchoring and stabilizing 9CDOT within the binding pocket. Compared to the standard, 9CDOT forms two π–π interactions with TRP111 and TRP219 engaging the first and third aromatic rings respectively, and a hydrogen bond is formed between LEU300 and the oxygen atom of the third aromatic ring (Figure 2), stabilizing the ligand core and enhancing overall binding energy. In addition, surrounding non-polar residues such as LEU300, VAL297, and ALA299 are likely to engage in hydrophobic and van der Waals interactions, which support ligand accommodation and contribute to binding affinity. The presence of two chlorine atoms in the ligand suggests potential halogen bonding interactions. One chlorine is oriented towards TRP219, indicating a possible π–π-halogen interaction, while the second is directed towards LEU300 and LEU301, suggesting halogen–hydrophobic interactions. These interactions collectively improve the specificity and affinity of 9CDOT within the binding site. The interactions of the reference inhibitor sulindac sulfone and 9CDOT with aldose reductase are illustrated in Figure 2 and summarized in Table 1.

**TABLE 1:** Molecular docking results showing Docking score, binding free energy and key active-site interactions.

Sl no	Compound name	Binding free energy (kcal/mol)	Docking score (kcal/mol)	No. of. H-bond	No. of. Pi-pi stacking interactions	Interacting residues
1	9-Carbomethoxy-6,11-dichloroxy-5-oxoxantho [3,2-g]tetralin (9CDOT)	−69.63	−8.492 kcal	1	2	LEU300, TRP219, TRP111
2	Sulindac sulfone (Standard)	−55.10	−9.95	2	1	TRP111, TYR48, PHE122

### 3.3 Toxicity prediction using ProTox3 webserver

ProTox3 was used to predict various toxicities, and the results were tabulated (Table 2). The analysis predicted that the phytocompound 9CDOT possesses a generally favorable toxicity profile. The compound was non-hepatotoxic, non-neurotoxic, non-carcinogenic, non-immunotoxic, non-mutagenic, non-cytotoxic, non-ecotoxic, non-nutritionally toxic, indicating low potential for systemic and environmental toxicity. The predicted LD_50_ of 9CDOT is 2000 mg/kg, and it belongs to Toxicity Class 4. Overall, these results suggest that 9CDOT is a safe candidate for further, *in silico* and experimental investigations.

**TABLE 2:** Predicted toxicity profile of 9CDOT obtained from ProTox3.

Target	Prediction	Probability
Hepatotoxicity	Inactive	0.66
Neurotoxicity	Inactive	0.82
Carcinogenicity	Inactive	0.52
Immunotoxicity	Inactive	0.93
Mutagenicity	Inactive	0.5
Cytotoxicity	Inactive	0.73
Ecotoxicity	Inactive	0.54
Nutritional toxicity	Inactive	0.51

### 3.4 Molecular dynamics simulations

MD simulation elucidates structural stability, molecular interactions, and integrity over a 200 ns period, offering insights into the binding and interaction of compounds within the binding cavity. To investigate dynamic stability, a detailed analysis of Apo protein, Aldose reductase-sulindac sulfone complex and Aldose reductase-9CDOT complex were subjected to MD simulation for 200 ns.

#### 3.4.1 Analysis of protein-ligand structural stability using RMSD

**FIGURE 3:**
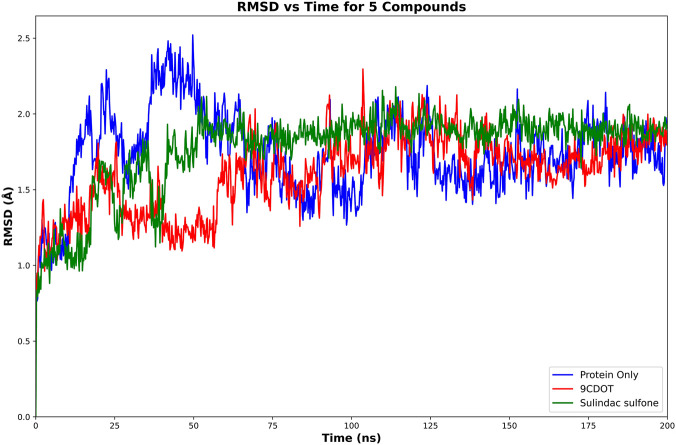
Cα RMSD plots of Aldose Reductase in complex with 9CDOT compared to the unbound (protein only) and Protein-Sulindac Sulfone (standard inhibitor) complex.

Throughout the 200 ns simulation, the structural stability and conformational integrity of protein-ligand complexes were assessed using the Root Mean Square Deviation (RMSD) analysis. In order to evaluate intrinsic stability, aldose reductase was first simulated at its apo form after the ligand was removed. This resulted in an average RMSD of 1.73 Å (Figure 3), and the standard inhibitor complex showed a similar RMSD value of 1.73 Å (Figure 3), indicating stable binding and minimal deviation from the initial structure. The 9CDOT-bound complex, on the other hand, showed a slightly reduced average RMSD of 1.58 Å (Figure 3), indicating structural stability and compactness.

#### 3.4.2 Analysis of residue flexibility using RMSF

**FIGURE 4:**
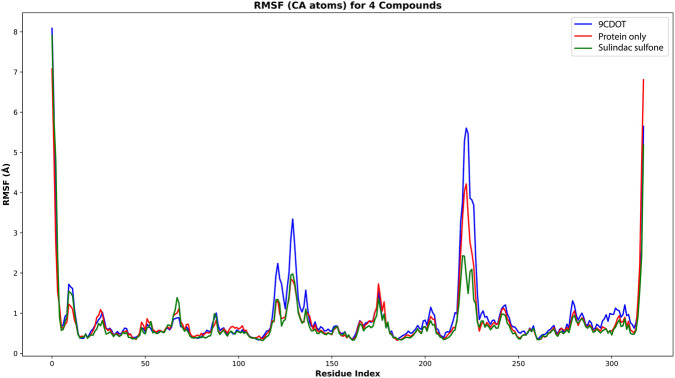
Illustrates the RMSF value of Cα throughout the 200 ns simulation period. The 9CDOT complex, Protein only and protein-Sulindac Sulfone complex is represented in blue, red and green colour respectively.

The flexibility of individual residues during the simulation was evaluated using the Root Mean Square Fluctuation (RMSF) analysis and the lower RMSF values indicate greater stability, whereas higher values signal reduced stability. The protein-9CDOT complex displayed a slightly greater fluctuation of 0.78 Å compared to the standard complex’s average RMSF of 0.73 Å (Figure 4). Throughout the 200 ns simulation, the overall low RMSF values across the binding site residues show limited conformational flexibility, indicating that ligand binding contributes to structural rigidity and overall complex stability.

#### 3.4.3 Protein ligand interactions

**FIGURE 5:**
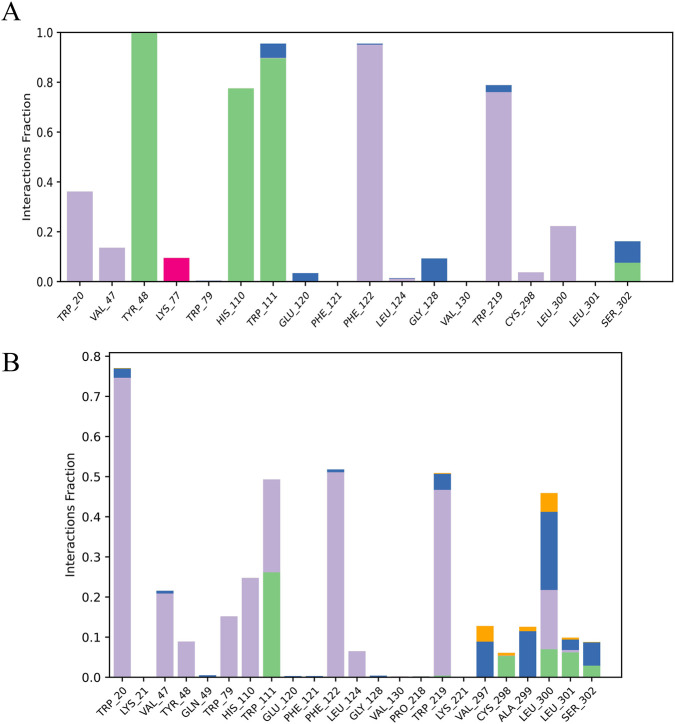
Protein-Ligand contact between **(A)** Sulindac sulfone (standard), **(B)** 9CDOT.

The analysis of the protein-ligand interactions between 9CDOT and sulindac sulfone to the ligand-protein binding site of Aldose Reductase revealed distinct interaction characteristics. Throughout the simulation, TRP111 maintains sustained connections in both standard and ligand complexes, according to the interaction histogram (Figure 5). In the 9CDOT complex, TRP111 alternates between hydrophobic contacts and hydrogen bonding, underscoring its crucial role in ligand stabilization, while in the conventional complex, it displays a stable hydrogen bond for almost half of the simulation duration.

For 9CDOT, residues TRP20, PHE122 and TRP129 predominantly exhibit hydrophobic interactions, with TRP20 maintaining these contacts consistently across the 200 ns simulation period. Overall TRP20, TRP111, PHE122, and TRP219 exhibited hydrophobic interactions exceeding 50% of total simulation time, indicating strong and stable ligand association. Furthermore, residues TRP111 demonstrated hydrogen bond formation in 25% and the other half as hydrophobic interaction of the entire duration. Additionally, residues VAL297, CYS298, ALA299, LEU300, LEU301, and SER302 exhibit multiple interactions, including hydrophobic interactions, and hydrogen bonds, halogen bonds and water bridge interactions. Suggesting their collective contribution to maintaining the structural stability of both standard and ligand complexes.

#### 3.4.4 Post MDS-MMGBSA-based binding free energy analysis

The estimation of binding free energy of ligand-Aldose reductase complexes provides insights into the stability and affinity of their interaction. For this purpose the top-binding ligand 9CDOT along with sulindac sulfone, was subjected to MM-GBSA analysis after the molecular dynamics simulation run. Sulindac sulfone exhibited a binding free energy of −122.63 kJ/mol, whereas 9CDOT showed a more favorable binding free energy of −129.45 kJ/mol (Table 3). These analyses demonstrated that 9CDOT possesses a stronger binding affinity and comparatively better binding free energy than the standard.

**TABLE 3:** The binding free energy along with contributing energies of the 9CD0T-Aldose reductase complex and Sulindac sulfone-Aldose reductase complex.

Title	Binding free energy (kcal/mol)	Van der waals energy (kcal/mol)	Coulomb energy	Lipophilic energy	H bond
9-Carbomethoxy-6,11-dichloroxy-5-oxoxantho [3,2-g]tetralin (9CDOT)	−129.45 ± 7.06	−82.08 ± 4.27	−39.80 ± 31.98	−21.80 ± 0.74	−14.66 ± 1.53
Sulindac sulfone (Std)	−122.63 ± 8.92	−84.87 ± 7.19	−15.05 ± 30.24	−21.09 ± 0.73	−15.43 ± 1.16

#### 3.4.5 Free energy landscape (FEL)

**FIGURE 6:**
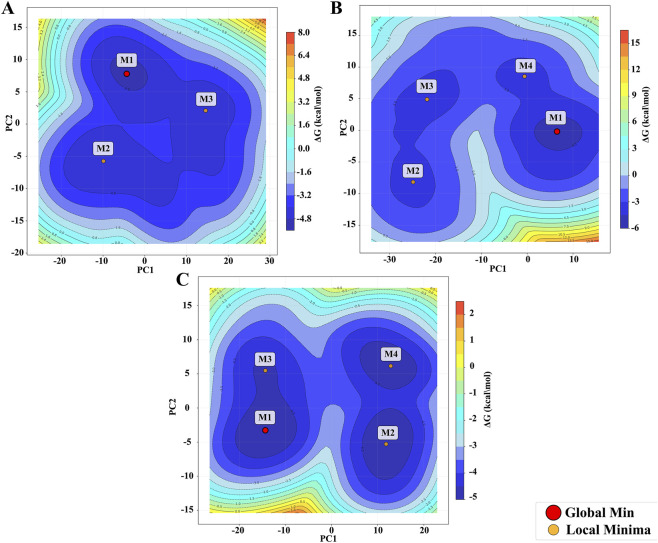
Free Energy Landscape (FEL) of **(A)** Protein-only, **(B)** Sulindac sulfone-protein complex, **(C)** 9CDOT-Protein complex.

Gibbs free energy landscape (FEL) was examined for the protein_only, the standard inhibitor complex, and the 9CDOT-protein complex, for the better understanding of the influence of ligand binding in protein dynamics. The conformation with lowest Gibbs free energy shows the stable structure that represents a favorable protein-ligand interaction. FEL describes the conformational stability and flexibility of protein during the simulation. According to (Figure 6) the deeper blue regions show the lower energy basins that indicate thermodynamically stable conformations. The apo protein displays a deeper global minima (M1) and a broad and relatively shallow energy basin with fewer distinct minima (M2, M3) suggests that the protein only structure itself is flexible and can have different conformations. The standard inhibitor complex has three local minima (M2, M3, M4) surrounding the main energy basin (M1). This suggests that it provides noticeable stabilization while still allowing the protein to maintain moderate flexibility. In contrast, 9CDOT-protein complex exhibits a single, deep and distinct minimum (M1), indicating that upon ligand binding protein takes on a stable and energetically favourable conformation.

These findings demonstrate that ligand binding has significance in the conformational landscape of protein. The 9CDOT-protein complex shows stronger conformational stability than the standard inhibitor complex that exhibits moderate stabilization and the apo protein which is flexible and less stable. The deep energy basin of 9CDOT-protein complex demonstrates it as an energetically favorable promising ligand that can keep the stable conformation of the protein.

### 3.5 Predictive performance and statistical evaluation of the PCA–PLS QSAR model

**FIGURE 7:**
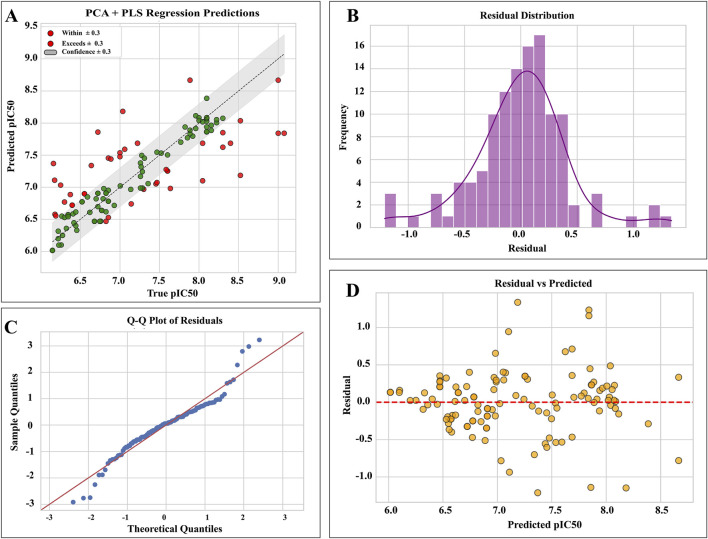
Statistical evaluation of the PCA–PLS QSAR model. **(A)** Correlation between predicted and experimental pIC_50_ values. **(B)** Indication of symmetric and near-normal error spread by residual distribution **(C)** Q–Q plot confirming normality of residue. **(D)** Residuals versus predicted pIC_50_ values.

The model was generated to predict the IC_50_ value of 9CDOT. A total of 119 compounds with 158 descriptors that converged into 20 PCA latent components with 95% descriptor variance and 5 were used as PLS latent components to maximize covariance between molecular descriptors and pIC_50_ values. The PCA and PLS regression model exhibited strong predictive capability, evidenced by a R^2^ value of 0.692, which signifies that roughly 69% of the variance in the experimental pIC_50_ values was explained by the model. The scatter plot (Figure 7A) depicting predicted and true pIC50 values indicates that the majority of predictions reside within a ±0.3 confidence interval, supported by low RMSE (0.4141) and MAE (0.2965) values.

With a small percentage of predictions exhibiting a large deviation from the observed data, the metrics indicate that the model can accurately estimate bioactivity. The residual histogram (Figure 7B) and Q-Q plot (Figure 7C) showed that the residuals had a symmetric distribution around zero with little deviation, confirming the accuracy of the model’s error structure and the absence of bias. The evaluation of residual diagnostics confirms the suitability of the model. The residuals against anticipated values plot (Figure 7D) verifies consistency by showing no systematic patterns or trends over the prediction range, whereas the Q-Q plot shows that most residuals follow normal expectations with only slight deviations at the extremes. With a maximum absolute error of 1.34 and a mean error near zero, the model shows unbiased residuals and no significant outliers. With prediction errors mostly falling within a reasonable margin and residuals meeting the fundamental statistical assumptions needed for regression analysis, the results show that the PCA + PLS regression method produces dependable pIC_50_ predictions. The 9CDOT has a predicted pIC_50_ of 7.52 (IC_50_ = 30 nM), indicating a very effective and potential therapeutic candidate.

## 4 Discussion

Various bioactive compounds and their therapeutic validations support the traditional application of *B. buxifolia* in treating diverse ailments (Selvi et al., 2017). Numerous phytochemicals, particularly flavonoids, have been identified as potential therapeutics against various detrimental diseases and infections. These plant derived chemicals are noteworthy for their considerable medicinal potential (Timotheous et al., 2025). Among these disorders diabetes mellitus is a polygenic metabolic condition which often requires a continuous pharmacological interventions through insulin therapy or other oral antidiabetic drugs such as biguanides, sulphonylureas, thiazolidinediones, DPP4 inhibitors, and α-glucosidase inhibitors (Chaudhary et al., 2022). Despite their efficacy, the long-term use of these agents is frequently limited by side effects and resistance, highlighting the importance of discovering novel, plant-derived inhibitors with improved safety profiles.

In this study, the co-crystallised ligand sulindac sulfone was redocked into the active site of aldose reductase to validate the docking analysis. The reliability of the docking parameters was confirmed by the RMSD value of 0.117 Å between the redocked and original ligand-protein complex. The minimal deviation here demonstrates the accuracy and reliability of the computational method in reproducing experimental confirmations, which is falling in the accepted RMSD threshold, of around 2.0 Å. This validation ensures that the following virtual screening of the potential inhibitors is both precise and robust (Agu et al., 2023; Hevener et al., 2009).

Virtual screening has become a powerful approach for identifying therapeutically potential compounds by combining molecular docking with molecular dynamics simulations (Azeem et al., 2022). Through this computational approach, 9-Carbomethoxy-6,11-dichloroxy-5-oxoxantho [3,2-g]tetralin (9CDOT) was identified as a potential inhibitor of aldose reductase with a docking score of −8.492 kcal/mol and a binding energy of −69.63 kcal/mol, indicating its strong affinity and stability. Although reference sulindac sulfone displayed a slightly higher docking score of −9.95 kcal/mol, its binding energy of −55.10 kcal/mol suggests comparatively less stable interaction. The comprehensive binding analysis revealed that the 9CDOT complex engaged in two *π*-*π* stacking interactions with TRP111 and TRP219 and a hydrogen bond with LEU300 within the active site of aldose reductase. These non-covalent interactions together improve the binding specificity and structural stability of the compound, supporting its potential as a strong inhibitor.

Molecular dynamics simulations of 200 ns were used to validate the dynamic stability of the ligand-protein complexes. The RMSD value of the apo form and standard inhibitor complex were similar at 1.73 Å, while the 9CDOT complex exhibited slightly lower fluctuations of 1.58 Å, indicating improved structural stability (Asnawi et al., 2024). The increased stability of the protein inhibitor complex supports the strong binding affinity predicted by docking studies. These findings were confirmed by the MM-GBSA calculation results, which revealed that the 9CDOT complex exhibited a more favorable binding free energy (−129.45 kJ/mol) compared to the sulindac sulfone complex (−122.63 kJ/mol), indicating a stronger and more energetically stable interactions. Throughout the 200 ns simulation, TRP111, TRP20, PHE122, and TRP219 consistently participated in maintaining the hydrophobic and hydrogen bond interactions according to the Residue level interaction analysis. For approximately 25% of the simulation period, TRP111 formed hydrogen bonds, and engaged in hydrophobic interactions for an additional 25%, underscoring its essential role in ligand stabilization. The additional non-covalent bonds, reinforcing overall structural stability, was contributed by other surrounding residues such as VAL297, CYS298, ALA299, LEU300, LEU301, and SER302. Minimal atomic fluctuations represented by the obtained RMSF values (0.78 Å) for 9CDOT and (0.73 Å) for the standard indicates a rigid complex structure and stable binding dynamics (Liu et al., 2025).

A PCA-PLS quantitative structure-activity relationship (QSAR) model was developed to predict the biological activity of the studied compounds in order to support the molecular modeling results. With an R2 of 0.692 and low error metrics of RMSE of 0.4141 and MAE of 0.2965, the model demonstrated high predictive capability. 9CDOT’s estimated pIC_50_ was 7.52, which translates to an IC_50_ of 30 nM, indicating that it is a very effective aldose reductase inhibitor. Thus, the integration of docking and molecular dynamics with AI-based QSAR modeling provides a strong framework for comprehending structure-activity connections and ranking promising compounds for more research.

The preliminary safety of 9CDOT was confirmed by the computational toxicity predictions derived from the ProTox3 webserver. The compound was predicted to have a favourable toxicology profile across vital organ systems, being non-cytotoxic, non-hepatotoxic, non-neurotoxic, non-carcinogenic (Zhang et al., 2025). A comprehensive *in silico* and *in vivo* validations will be crucial to confirm these predictions, optimize the therapeutic window and investigate structural modifications to mitigate any negative effects as computational toxicity assessment provides only an initial risk indication.

## 5 Conclusion

This study highlights the antidiabetic potential of *Barleria buxifolia L*, a plant traditionally used by ethnic communities in the southern Western Ghats. This study offers molecular-level evidence in favour of the ethnomedicinal use of human aldose reductase, a crucial enzyme associated with developing and progressing diabetic problems such as neuropathy and retinopathy. Among the bioactive phytocompounds that were examined, 9-Carbomethoxy-6,11-dichloroxy-5-oxoxantho [3,2-g]tetralin showed excellent stability and positive docking results in molecular dynamics simulations, making it a prospective inhibitor. The phytocompound’s potential role as an active principle in *B. buxifolia* was confirmed by the fact that its post-MD binding free energy of −129.45 kJ/mol was higher than that of the conventional sulindac sulfone (−122.63 kJ/mol). Strong specificity and affinity for aldose reductase are demonstrated by multiple hydrogen bonds, π-π stacking, hydrophobic contacts, and halogen bonding with critical residues. These investigations suggest that the phytocompound could be a promising starting molecule for developing novel antidiabetic medications. Additionally, this predicted data highlights how important it is to combine computational methods with traditional wisdom in drug discovery. The strong inhibitory potential of the compound 9CDOT was confirmed by the high predictive performance (R^2^ = 0.692) of AI-driven PCA-PLS QSAR model, which displayed a predicted pIC_50_ value of 7.52 (equivalent to an IC_50_ of 30 nM). This integrated application of AI modeling with molecular docking and dynamics offers a solid framework for identifying and prioritizing lead compounds for further investigation. Nevertheless, extensive preclinical and clinical research is necessary to substantiate these findings, ultimately establishing *B. buxifolia* as a promising natural source for antidiabetic drug development.

## Data Availability

All data generated or analyzed during this study are publicly available.
