# Supplementary material for: Machine learning-based QSAR and molecular modeling of phytocompounds in Barleria buxifolia L. as a potential aldose reductase inhibitor
Source: Front Bioinform. 2026 Mar 3;6:1766339. doi: 10.3389/fbinf.2026.1766339 (PMC12992281; doi:10.3389/fbinf.2026.1766339)
Supplement: Supplementary file 1 [file Table1.docx]

**Supplementary Table I. Identification of bioactive vital compounds in methanolic aerial extract of *Barleria buxifolia.***

| **S. No** | **Name of the compound** | **Molecular**  **formula** | **Structure** |
| --- | --- | --- | --- |
| 1. | Oxalic acid, cyclohexylmethyl ethyl ester | C_11_H_18_O_4_ | 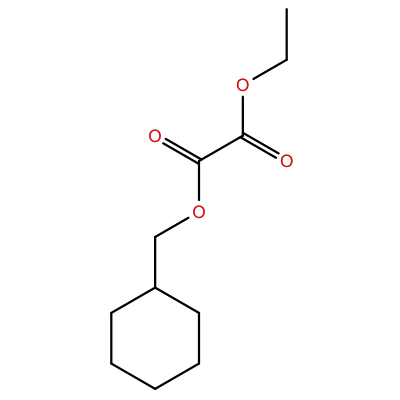 |
| 2. | 4-thiatricyclo [5.4.0.0(2,6)] undecan-8-one 4,4-dioxide | C_10_H_14_O_3_S | 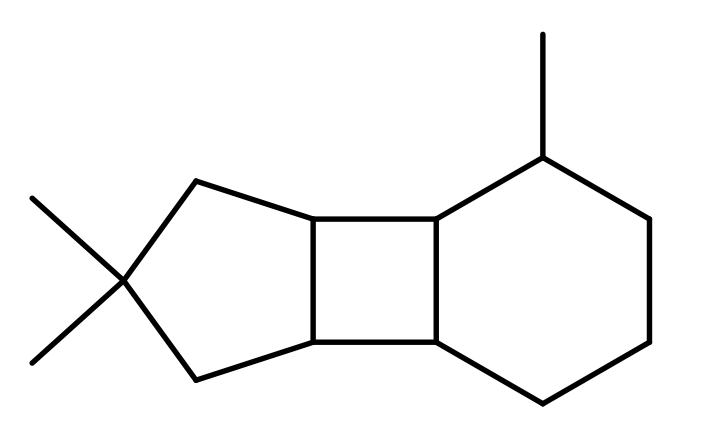 |
| 3. | 3-(Acetyloxy)-cis-1,2-epoxycyclohexane | C_8_H_12_O_3_ | 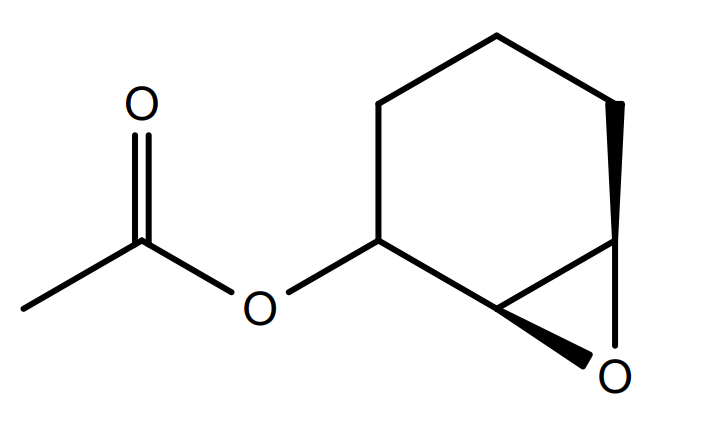 |
| 4. | (2R,3R,4S)-3-dimethyl-t-butylsiloxy-2,4-dimethylhexanal | C_14_H_30_O_2_Si | 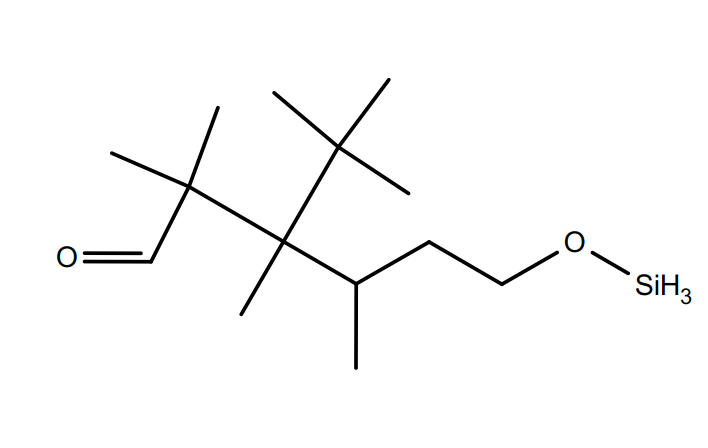 |
| 5. | 2-methyl-1,2,3,4-tetrahydroisochinolin-8-amin | C_10_H_14_N_2_ | 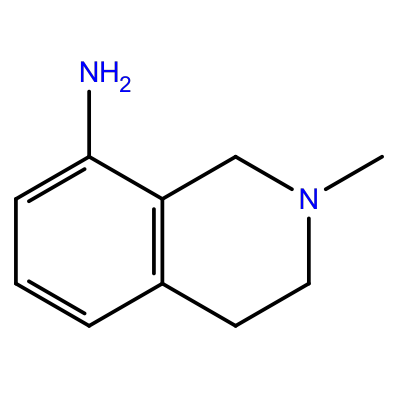 |
| 6. | Methyl 6-(vinylidene) penta-2,4-dien-1-oate] | C_9_H_10_O_2_ | 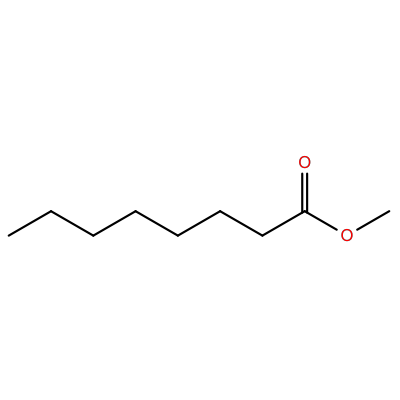 |
| 7. | (2S,3S)-2-(hydroxymethyl)-2-methyltetrahydro-2H-thiopyran-3-ol | C_7_H_14_O_2_S | 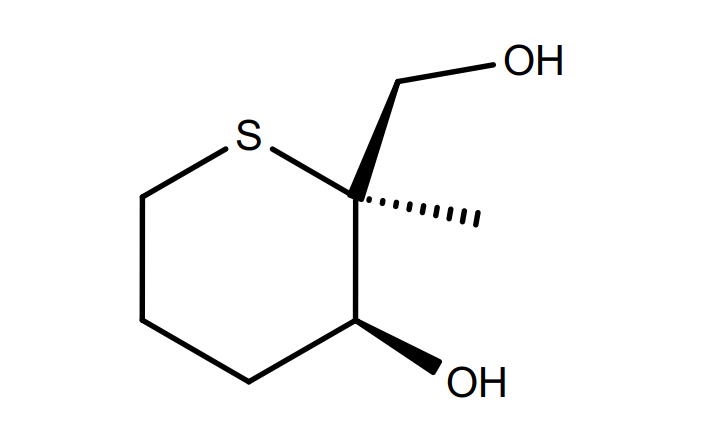 |
| 8. | (+-)-4-ethoxy-5-methyl-2,5-dihydrofuran-2-one | C_7_H_10_O_3_ | 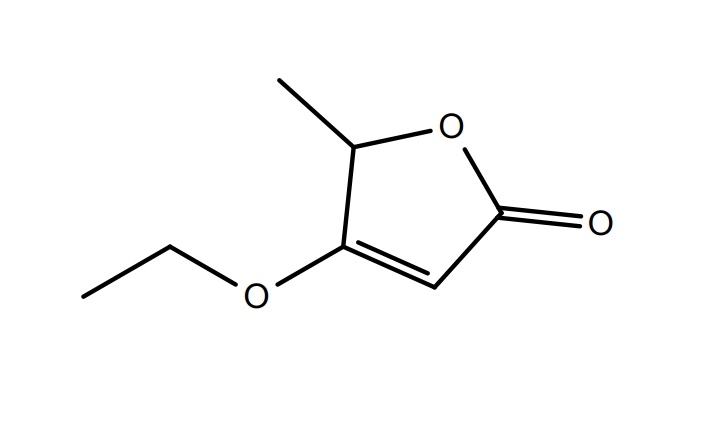 |
| 9. | 1,3-D5-hexan-2-one  2,4 dinitrophenylhydrazone | C_12_H_11_D_5_N_4_O_4_ | 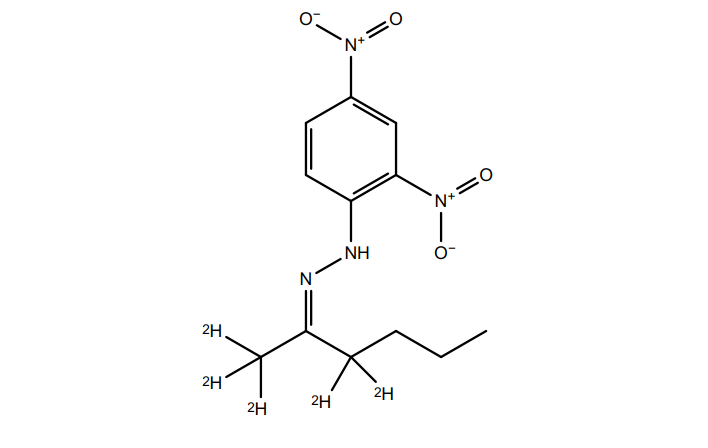 |
| 10. | S-Methyl 2-pyridyldithiocarboxylate | C_7_H_7_NOS | 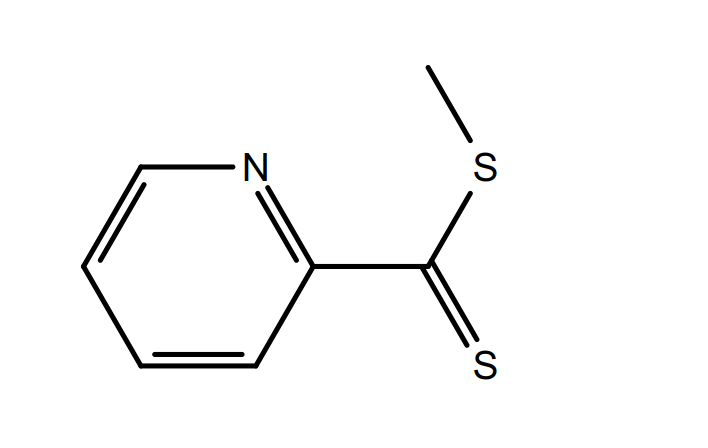 |
| 11. | (E)-1-Bromo-7-(3,5-dimethoxyphenyl)-4-methyl-4-heptene | C_16_H_23_BrO_2_ | 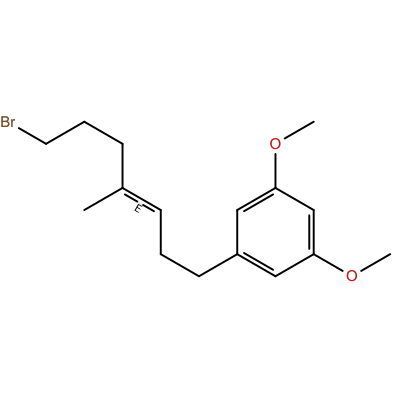 |
| 12. | Megastigmatrienone | C_13_H_18_O | 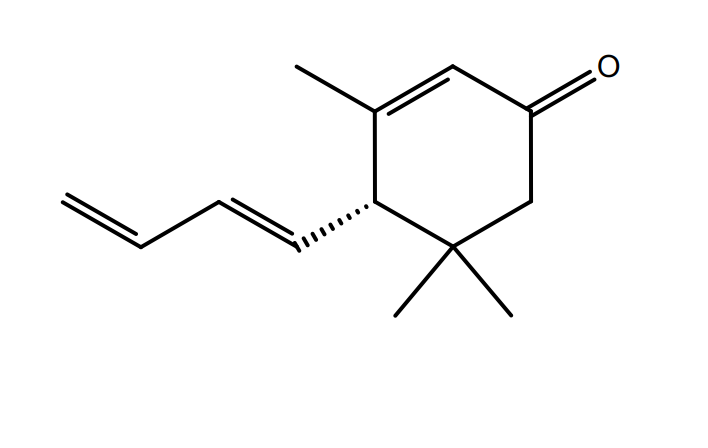 |
| 13. | Benzyl geranyl carbonate | C_18_H_24_O_3_ | 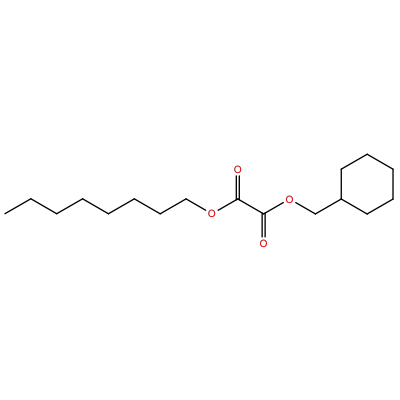 |
| 14. | 4,4,5,8-tetramethylchroman-2-ol | C_13_H_18_O_2_ | 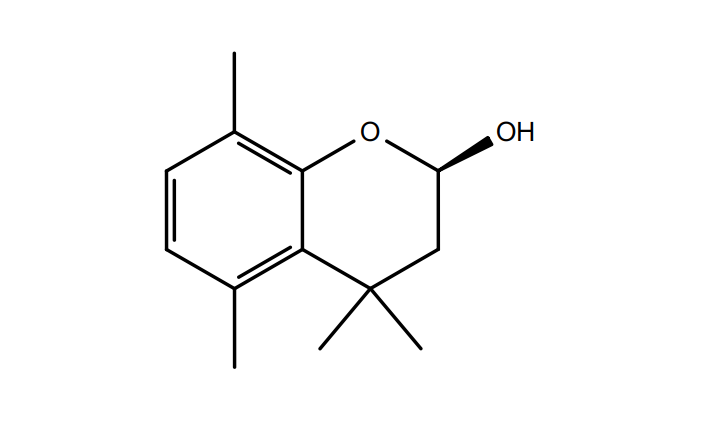 |
| 15. | Iron,  dicarbonyl(ü5-2,4-cyclopentadien-1-yl)(2-pyridinylmethy  l)- (CAS) | C_13_H_11_FeN  O_2_ | 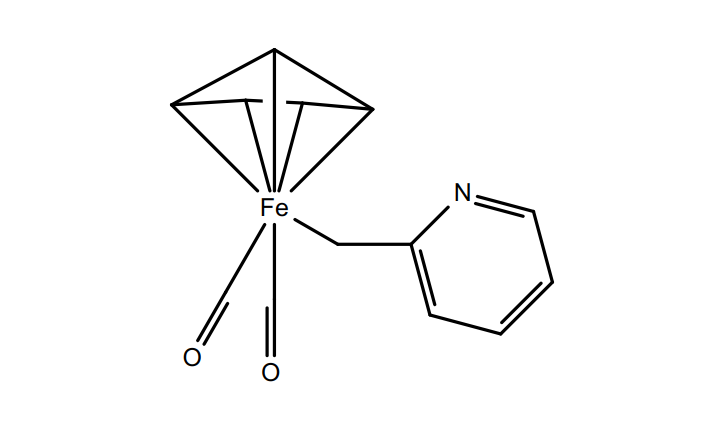 |
| 16. | Hexadecanoic acid, methyl ester (CAS) | C_17_H_34_O_2_ | 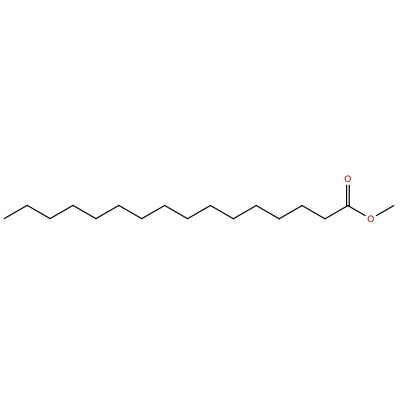 |
| 17. | 4-Hydroxy-4-(1'-methoxy-1'-cyclopropyl)-3,3,5,8,10,10-  hexamethyltricyclo[6.2.2.0(2,7)]dodeca-5,11-diene-9-one | C_22_H_32_O_3_ | 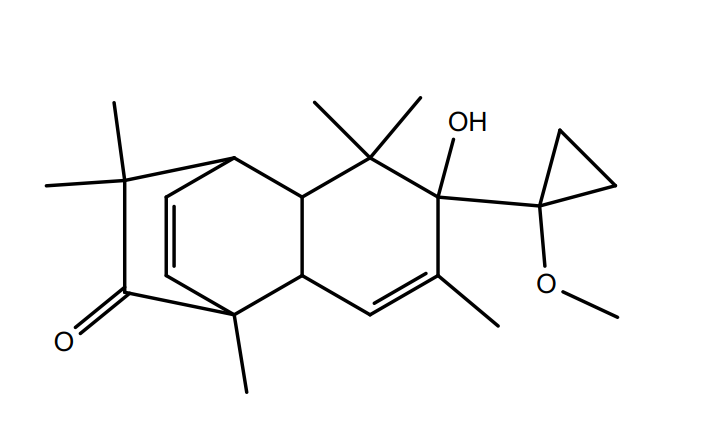 |
| 18. | 2,4,4-Trimethylcyclopenten-3-one | C_8_H_12_O | 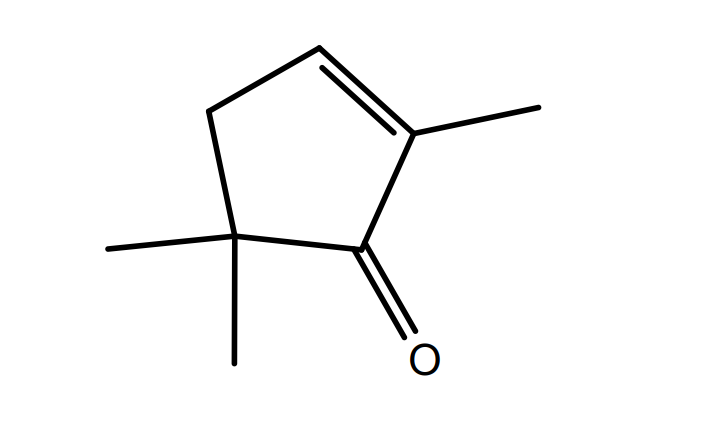 |
| 19. | 4,14-Dibromo(2.2)metacyclophane | C_16_H_14_Br_2_ | 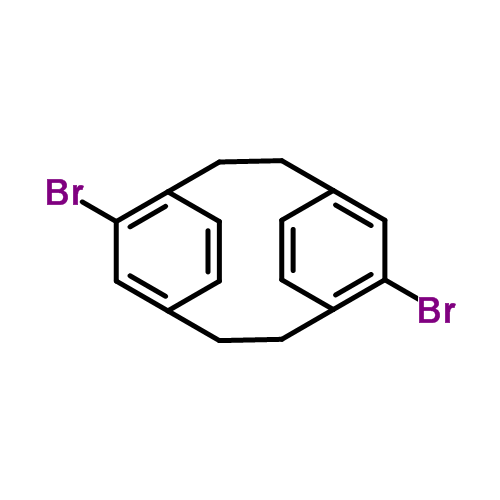 |
| 20. | 2-Hexadecen-1-ol, 3,7,11,15-tetramethyl-,  [R-[R*,R*-(E)]]- (CAS) | C_20_H_40_O | 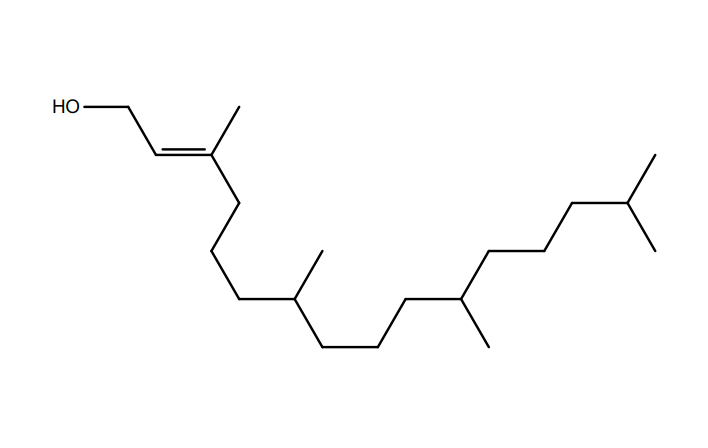 |
| 21. | 12-Tridecynoic acid, methyl ester (CAS) | C_14_H_24_O_2_ | 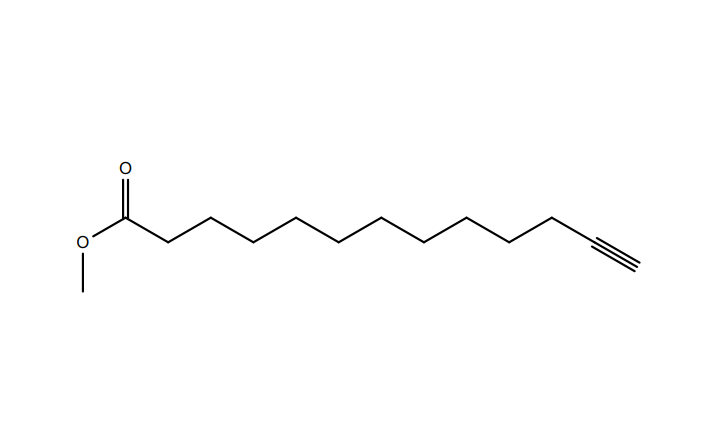 |
| 22. | 9,12,15-Octadecatrienoic acid, methyl ester, (Z,Z,Z)-  (CAS) | C_19_H_32_O_2_ | 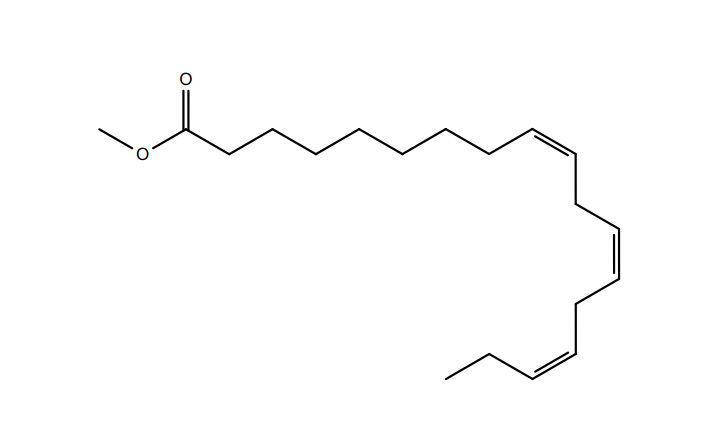 |
| 23. | N-Methyl-4-nitrobenzo-13-aza-1,4,7,10-tetraoxa-15-crown-5 | C_15_H_22_N_2_O_6_ | 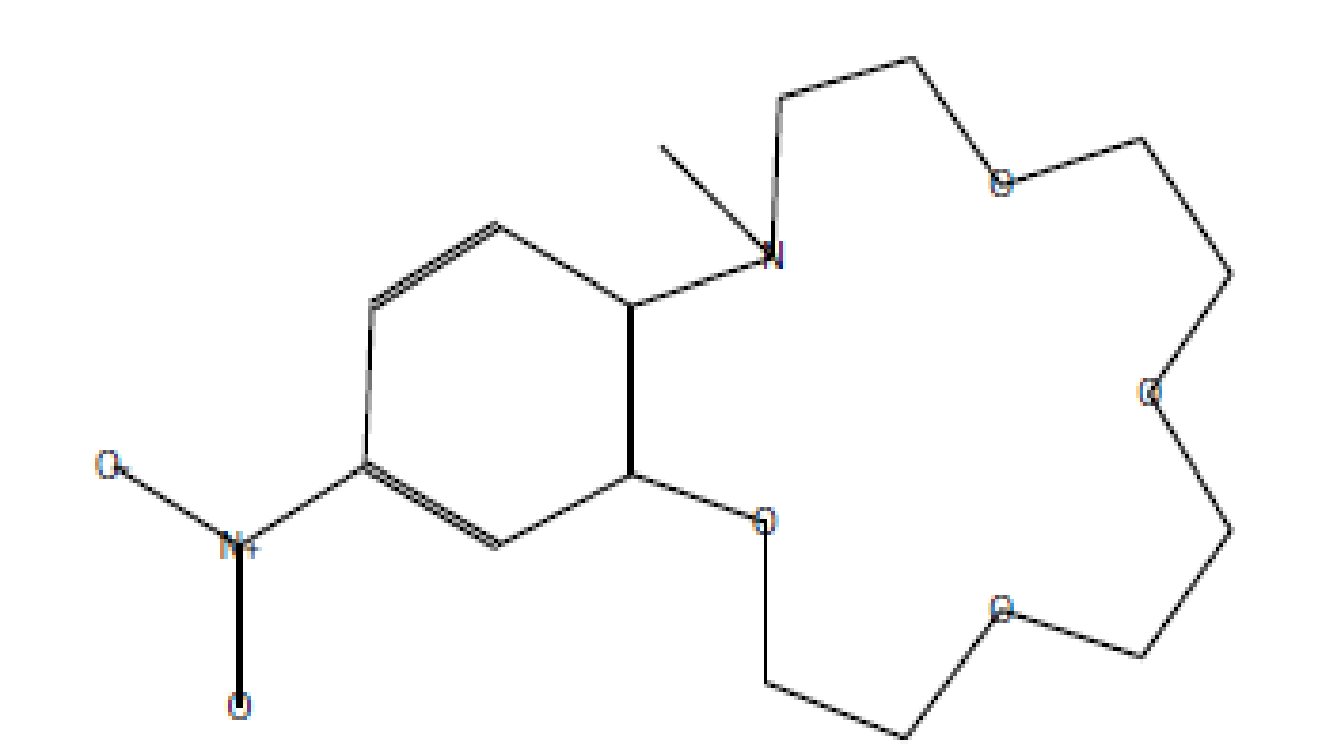 |
| 24. | 5-Hydroxy-6-methyl-12,13-dioxa-tricyclo[7.3.1.0(1,6)]tri decane-10-carboxylic acid, methyl ester | C_14_H_22_O_5_ | 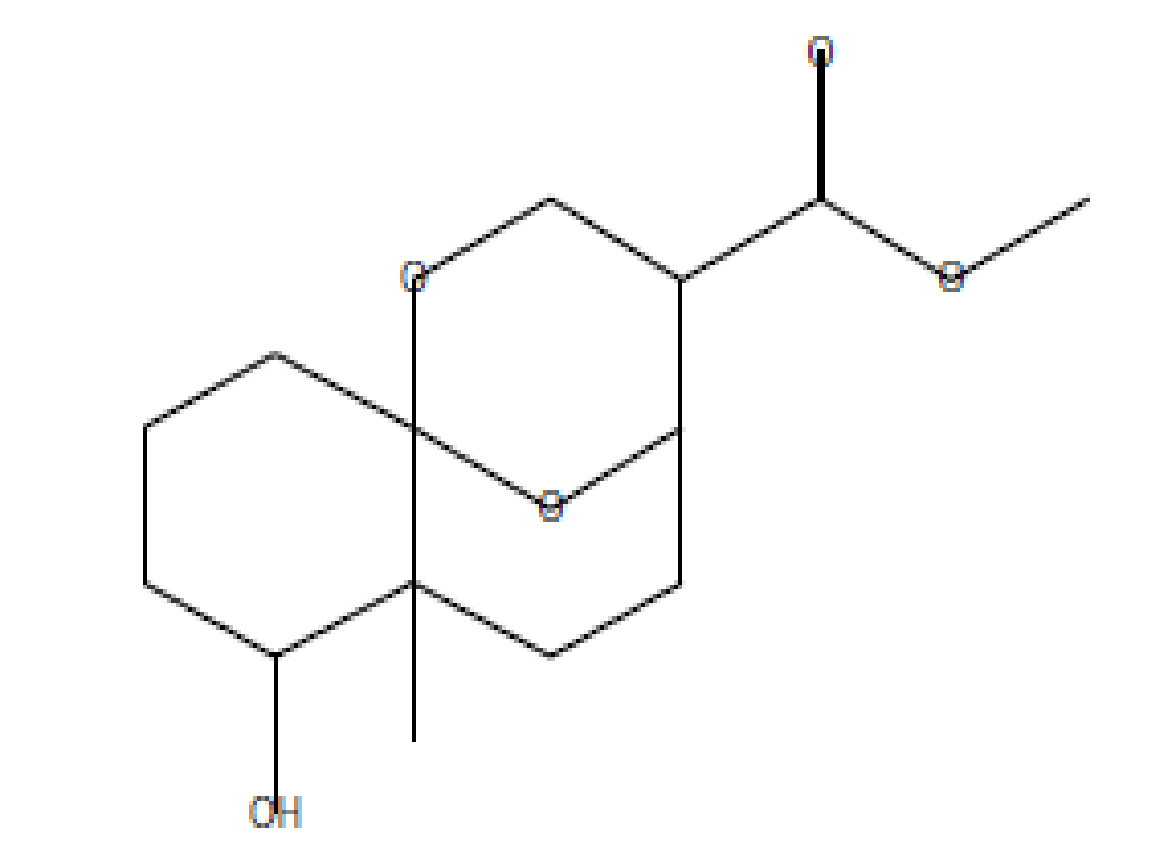 |
| 25. | 9-Carbomethoxy-6,11-dichloroxy-5-oxoxantho[3,2-g]tetralin | C_19_H_14_C_l2_O_6_ | 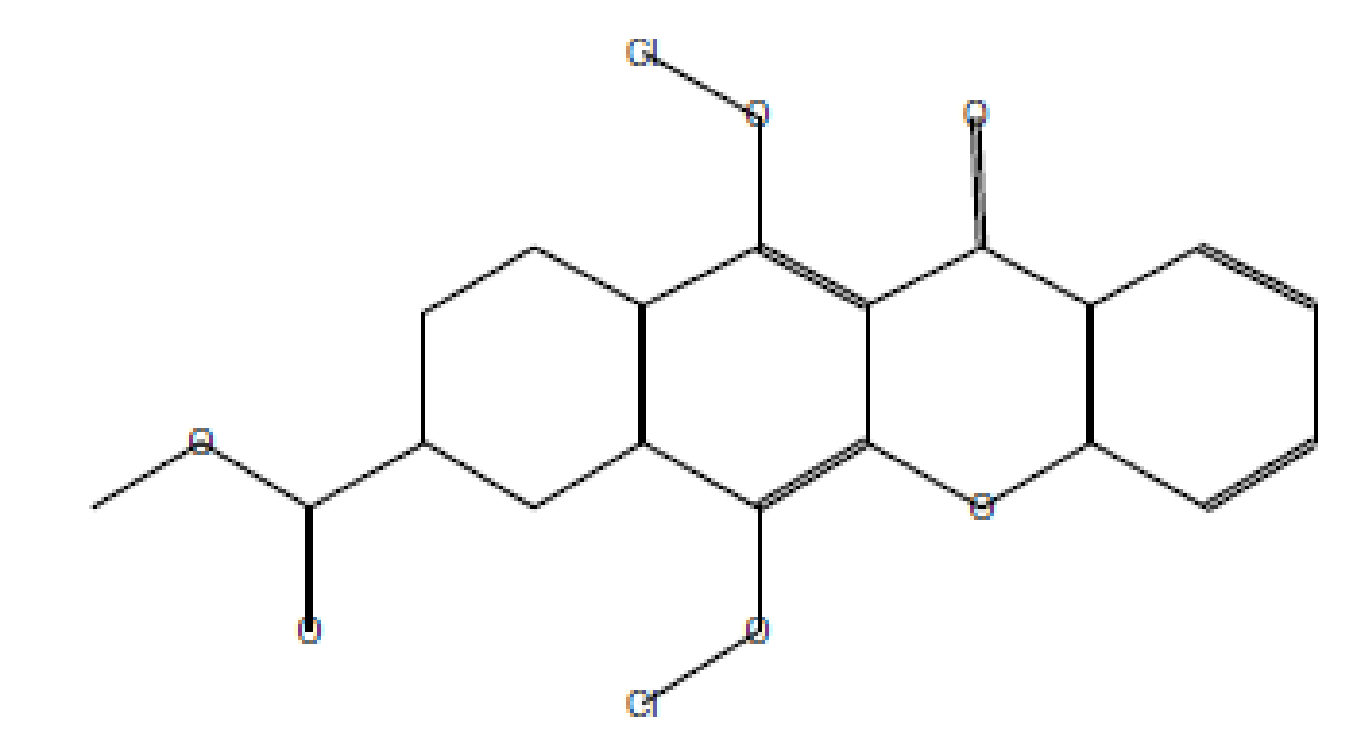 |
| 26. | 2-(4-(4-chlorophenyl)-1-oxo-2(1H)-phthala  zinyl)-N-(2,2,6,6 tetramethyl-4-piperidinyl) acetamide | C_25_H_29_C_l_N_4_O_2_ | 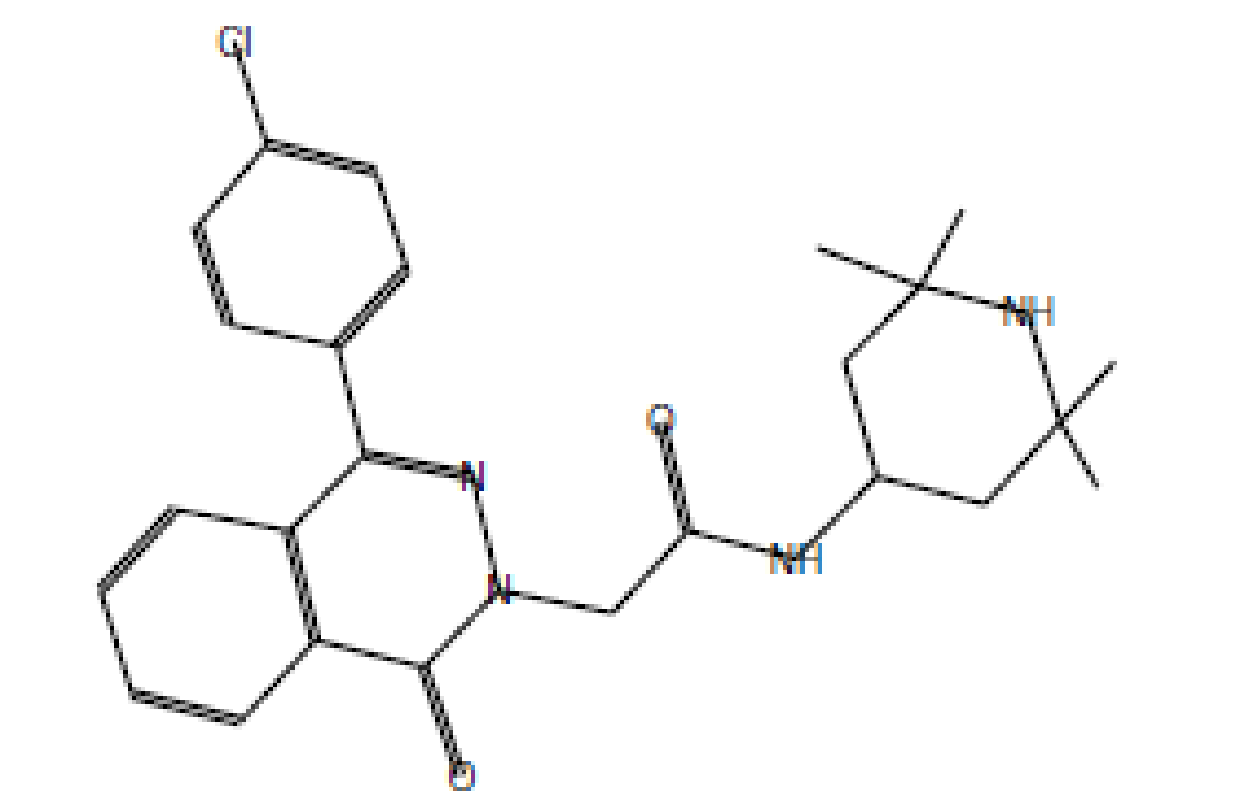 |
| 27. | 4-(4-Methoxy-6-methyl-5,6,7,8-tetrahydro-[1,3]dioxolo[4  ,5-g]isoquinolin-5-yl)-5-methyl-2,4-dihydro-pyrazol-3-one | C_16_H_19_N_3_O_4_ | 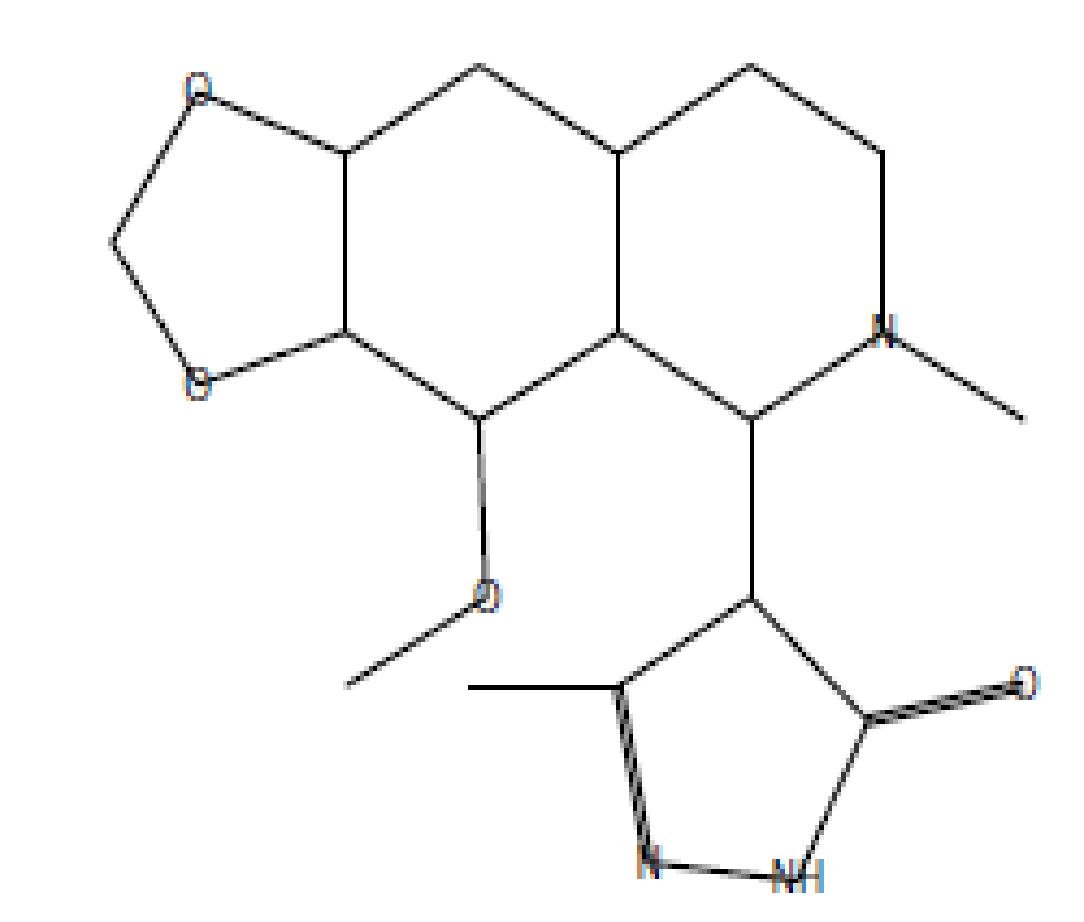 |
| 28. | 1,2-Benzenedicarboxylic acid, bis(2-ethylhexyl) ester(CAS) | C_24_H_38_O_4_ | 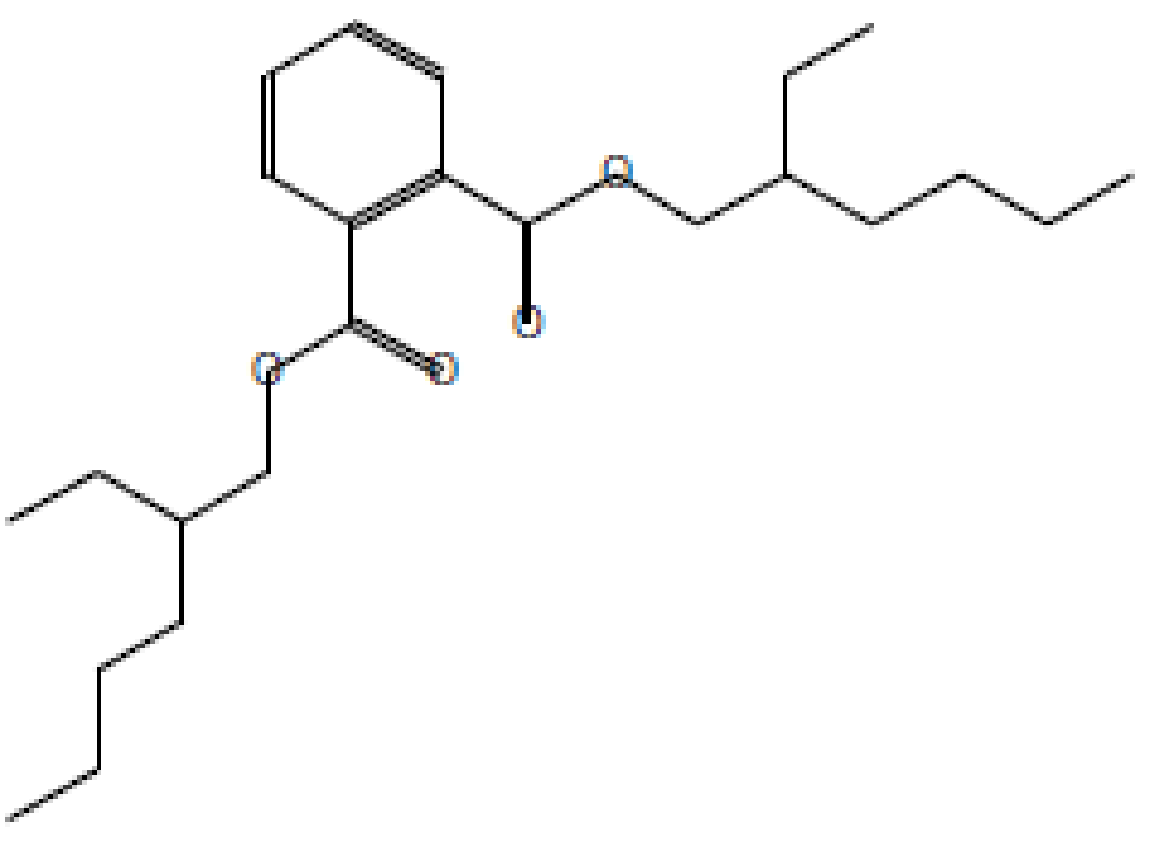 |
| 29. | 2,6,10,14,18,22-Tetracosahexaene,  2,6,10,15,19,23-hexamethyl- (CAS) | C_30_H_50_ | 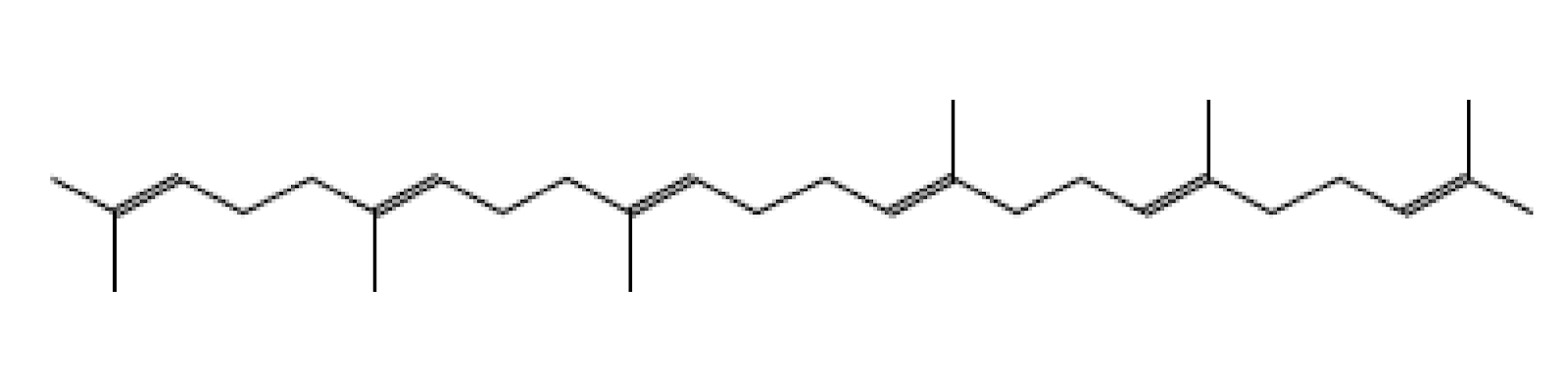 |
| 30. | anti-2,23-Dithia[3.3](1,4)triphenylenophane | C_40_H_28_S_2_ | 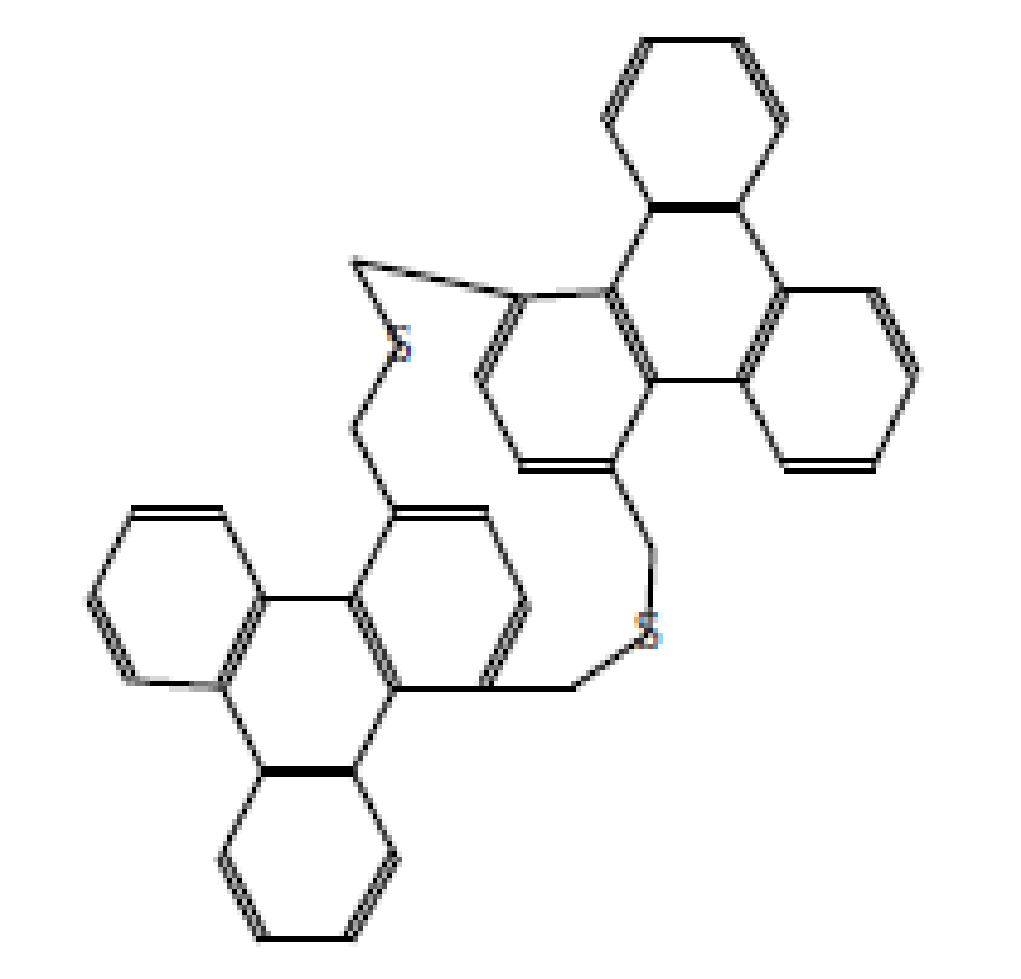 |

**Supplementary Table II.** ADME properties of screened phytocompounds against aldose reductase

| **Phytocompounds** | **CNS** | **Rule Of Five** | **Glide gscore** | **Pre MD - MMGBSA**  **(kcal/mol)** | **PSA** | **% Human Oral Absorption** | **QPPCaco** | **QPPMDCK** | **QPlogBB** | **QPlogHERG** | **QPlogKp** | **QPlogPo/w** | **QPlogPw** | **SASA** | **HBA** |
| --- | --- | --- | --- | --- | --- | --- | --- | --- | --- | --- | --- | --- | --- | --- | --- |
| 9-Carbomethoxy-6,11- dichloroxy-5-oxoxantho[3,2- g]tetralin | 0 | 0 | -8.49 | -69.63 | 86.07 | 100 | 837.25 | 1877.11 | -0.428 | -5.26 | -2.64 | 3.54 | 8.59 | 629.82 | 6 |
| 1,2-Benzenedicarboxylic acid, bis(2-ethylhexyl) ester(CAS) | -2 | 1 | -7.15 | -68.35 | 61.14 | 100 | 2158.78 | 1136.55 | -1.023 | -5.60 | -0.94 | 6.47 | 3.92 | 781.69 | 4 |
| (E)-1-Bromo-7-(3,5-  dimethoxyphenyl)-4-methyl- 4-heptene | 1 | 1 | -7.71 | -65.48 | 16.40 | 100 | 9906.04 | 10000 | 0.38 | -4.98 | -0.42 | 5.56 | 1.55 | 608.17 | 1.5 |
| 2,6,10,14,18,22-  Tetracosahexaene, 2,6,10,15,19,23-hexamethyl- (CAS) | 2 | 1 | -4.20 | -64.73 | 0 | 100 | 9906.04 | 5899.29 | 1.849 | -5.11 | 0.06 | 12.13 | - 4.40 | 867.66 | 0 |

| Oxalic acid, cyclohexylmethyl ethyl ester | -2 | 0 | -6.15 | -64.26 | 71.76 | 100 | 850.56 | 415.31 | -1.324 | -5.35 | -2.63 | 4.39 | 3.94 | 714.55 | 4 |
| --- | --- | --- | --- | --- | --- | --- | --- | --- | --- | --- | --- | --- | --- | --- | --- |
| anti-2,23-  Dithia[3.3](1,4)triphenylenop hane | 0 | 2 | -7.86 | -63.8 | 0 | 100 | 9906.04 | 10000 | -0.06 | -6.51 | 0.54 | 8.50 | 6.50 | 746.88 | 1 |
| 2-Hexadecen-1-ol, 3,7,11,15- tetramethyl | -1 | 1 | -4.73 | -59.8 | 22.90 | 100 | 2862.16 | 1541.63 | -0.92 | -5.30 | -1.18 | 6.37 | 2.03 | 748.31 | 1.7 |
| Megastigmatrienone | 0 | 0 | -8.03 | -57.48 | 28.72 | 100 | 3042.30 | 1646.77 | -0.01 | -3.63 | -2.06 | 3.05 | 3.22 | 454.85 | 2 |
| 12-Tridecynoic acid, methyl ester (CAS) | -1 | 0 | -4.92 | -56.77 | 37.89 | 100 | 2576.43 | 1375.98 | -0.761 | -5.21 | -1.40 | 4.39 | 2.26 | 610.47 | 2 |
| 9,12,15-Octadecatrienoic acid, methyl  ester, (Z,Z,Z)- (CAS) | -1 | 1 | -7.27 | -55.08 | 36.56 | 100 | 3029.63 | 1639.36 | -0.832 | -5.52 | -1.08 | 6.16 | 1.30 | 729.65 | 2 |
| 4-(4-Methoxy-6-methyl- 5,6,7,8-tetrahydro- [1,3]dioxolo[4  ,5-g]isoquinolin-5-yl)-5-  methyl-2,4-dihydro-pyrazol-3- one | 1 | 0 | -8.59 | -53.64 | 82.68 | 80.68 | 296.11 | 146.86 | 0.062 | -3.93 | -5 | 1.62 | 9.59 | 499.91 | 5.25 |
| 1,3-D5-hexan-2-one-2,4 dinitrophenylhydrazone | -2 | 0 | -5.87 | -51.14 | 116.66 | 79.72 | 151.25 | 64.22 | -1.742 | -4.68 | -3.9 | 2.35 | 6.73 | 552.78 | 4 |

| Hexadecanoic acid, methyl ester (CAS) | -1 | 1 | -3.70 | -47.58 | 36.17 | 100 | 2899.90 | 1563.61 | -0.926 | -5.38 | -1.21 | 5.81 | 0.71 | 719.56 | 2 |
| --- | --- | --- | --- | --- | --- | --- | --- | --- | --- | --- | --- | --- | --- | --- | --- |
| (+-)-4-ethoxy-5-methyl-2,5- dihydrofuran-2-one | 0 | 0 | -5.97 | -45.75 | 49.78 | 89.32 | 2068.44 | 1085.23 | -0.112 | -2.90 | -2.48 | 0.51 | 4.59 | 342.31 | 3.75 |
| 3-(Acetyloxy)-cis-1,2- epoxycyclohexane | 1 | 0 | -7.22 | -42.69 | 49.40 | 95.04 | 3648.83 | 2004.34 | 0.162 | -2.41 | -2.26 | 0.74 | 4.59 | 351.17 | 4 |
| Methyl 6-(vinylidene) penta- 2,4-dien-1-oate] | 0 | 0 | -5.79 | -42.05 | 37.91 | 100 | 2577.06 | 1376.34 | -0.376 | -4.06 | -2.08 | 2.66 | 1.90 | 465.42 | 2 |
| hexamethyltricyclo[6.2.2.0(2, 7)]dodeca-5,11-diene-9-one | 1 | 0 | -8.35 | -41.77 | 42.05 | 100 | 3482.73 | 1905.90 | 0.04 | -3.56 | -2.02 | 4.55 | 6.28 | 583.67 | 3.5 |
| (2S,3S)-2-(hydroxymethyl)-2- methyltetrahydro-2H-  thiopyran-3-ol | 0 | 0 | -6.3 | -40.54 | 42.22 | 88.67 | 1642.73 | 1256.3 | -0.161 | -2.49 | -2.74 | 0.71 | 7.25 | 355.03 | 3.9 |
| 4-thiatricyclo [5.4.0.0(2,6)] undecan-8-one 4,4-dioxide | 0 | 0 | -7.58 | -40.04 | 70.04 | 74.57 | 498.381 | 239.46 | -0.5 | -2.75 | -4.04 | -0.11 | 7.53 | 405.8 | 6 |
| S-Methyl 2-  pyridyldithiocarboxylate | 2 | 0 | -5.4 | -39.01 | 21.01 | 100 | 6082.57 | 10000 | 0.485 | -3.84 | -1.13 | 2.23 | 3.99 | 371.47 | 2.5 |
| 5-Hydroxy-6-methyl-12,13- dioxa-tricyclo[7.3.1.0(1,6)]tri decane-10-carboxylic acid, methyl ester | 0 | 0 | -7.63 | -38.75 | 61.93 | 100 | 2117.15 | 1112.88 | -0.127 | -3.00 | -2.63 | 2.02 | 7.5 | 478.72 | 5.2 |

| 2,4,4-Trimethylcyclopenten-3- one | 1 | 0 | -6.25 | -26.78 | 25.25 | 100 | 4608.53 | 2579.78 | 0.296 | -2.77 | -2.03 | 1.71 | 3.01 | 343.98 | 2 |
| --- | --- | --- | --- | --- | --- | --- | --- | --- | --- | --- | --- | --- | --- | --- | --- |
| 4,14-  Dibromo(2.2)metacyclophane | 2 | 1 | -2.84 | -23.7 | 0 | 100 | 9906.04 | 10000 | 0.516 | -3.01 | -0.95 | 5.04 | 1.88 | 432.78 | 0 |
| (2R,3R,4S)-3-dimethyl-t- butylsiloxy-2,4-  dimethylhexanal | -1 | 0 | -8.52 | -23.22 | 33.15 | 100 | 3937.18 | 2176.08 | -0.241 | -2.59 | -1.53 | 3.10 | 6.61 | 486.06 | 2.8 |
